# Response Surface Study on Molecular Docking Simulations of Citalopram and Donepezil as Potent CNS Drugs

**DOI:** 10.22037/ijpr.2020.113644.14409

**Published:** 2021

**Authors:** Radin Alikhani, Ahmad Ebadi, Pari Karami, Sara Shahbipour, Nima Razzaghi-Asl

**Affiliations:** a *Students Research Committee, School of Pharmacy, Ardabil University of Medical Sciences, Ardabil, Iran. *; b *Department of Medicinal Chemistry, School of Pharmacy, Hamadan University of Medical Sciences, Hamadan, Iran. *; c *Medicinal Plants and Natural Products Research Center, Hamadan University of Medical Sciences, Hamadan, Iran. *; d *Biosensor Sciences and Technologies Research Center, Ardabil University of Medical Sciences, Ardabil, Iran. *; e *Department of Medicinal Chemistry, School of Pharmacy, Ardabil University of Medical Sciences, Ardabil, Iran. *; f *Pharmaceutical Sciences Research Center, Aradabil University of Medical Sciences, Ardabil, Iran. *

**Keywords:** Central nervous system, Citalopram, Donepezil, Binding, Target, Response Surface

## Abstract

Computer-aided drug design provides broad structural modifications to evolving bioactive molecules without an immediate requirement to observe synthetic restraints or tedious protocols. Subsequently, the most promising guidelines with regard to synthetic and biological resources may be focused on upcoming steps. Molecular docking is common *in-silico* drug design techniques since it predicts ligand-receptor interaction modes and associated binding affinities. Current docking simulations suffer serious constraints in estimating accurate ligand-receptor binding affinities despite several advantages and historical results. Response surface method (RSM) is an efficient statistical approach for modeling and optimization of various pharmaceutical systems. With the aim of unveiling the full potential of RSM in optimizing molecular docking simulations, this study particularly focused on binding affinity prediction of citalopram-serotonin transporter (SERT) and donepezil-acetyl cholinesterase (AChE) complexes. For this purpose, Box-Behnken design of experiments (DOE) was used to develop a trial matrix for simultaneous variations of AutoDock4.2 driven binding affinity data with selected factor levels. Responses of all docking trials were considered as estimated protein inhibition constants with regard to validated data for each drug. The output matrix was subjected to statistical analysis and constructing polynomial quadratic models. Numerical optimization steps to attain ideal docking accuracies revealed that more accurate results might be envisaged through the best combination of factor levels and considering factor interactions. Results of the current study indicated that the application of RSM in molecular docking simulations might lead to optimized docking protocols with more stable estimates of ligand-target interactions and hence better correlation of *in-silico* *in-vitro* data.

## Introduction

Mental disorders include a wide range of common neurological and psychiatric illnesses. The nature of CNS disorders changes across the human lifespan (1, 2) and affects a huge amount of people worldwide (3). Mental and neurological disorders pose the largest health, economic and social capital burden worldwide of any disease group. Indeed, the proportionate share of the total global burden of neurologic disorders is projected to rise, highlighting an urgent need for more selective and potent drugs to treat CNS disorders (1). The process of drug development is challenging, time-consuming, expensive, and requires tedious steps. To overcome these problems to some extent, *in-silico* drug design approaches seem to be cost- and time-efficient procedures. In this regard, valuable information about certain CNS targets and their interacted ligands/drugs is achievable via relevant online databases. On the basis of such data sources, molecular modeling studies aiming at structural elucidation of ligand-receptor interactions could be well established and developed. The final goal of such structure-based *in-silico* studies would be to accurately and precisely predict the interactions of candidate small molecules within desired target binding sites. According to this, molecular docking is the most popular virtual structure-based method since it predicts spatial pose(s) of ligands inside the binding site of docked receptors while at the same time estimates the affinity toward the macromolecular target(s) (4). Obtained information from the docking technique is useful for attaining drug-macromolecule complexes with optimized conformations and less binding free energy (5). Search algorithm and scoring function are principal components of different docking methods. Commonly utilized search algorithms are genetic algorithm, Monte Carlo, fragment-based and molecular dynamics within a popular docking package such as DOCK (6), AutoDock (7), Gold (8), FlexX (9), and Glide (10). There are different approaches to molecular docking procedures, which are mainly categorized as rigid ligand/rigid target, flexible ligand/rigid target, and flexible ligand/rigid target. However, a few docking packages provide flexible ligand/flexible target via allowing some kinds of protein flexibilities such as side-chain movements. 

Within recent advancements and regarding the important role of computational modeling in drug design, more correlated *in-silico in-vitro*/*in-vivo *experimental data has a determinant significance. More valid *in-silico* data with higher confidence intervals might be envisaged through careful inspection/optimization of the effective factors and their interactions on the final response. Traditional optimization methods consider the variation of one factor while holding others constant. It has been revealed that such customary techniques require more trial runs and are exclusively focused on the effect of just varied factors. This is a major technical bottleneck since the interferences among factors are not taken into account. Moreover, when multiple methodological factors are involved in a typical procedure, this technique becomes unproductive and time-consuming. Response surface methods (RSM) have been established to study factors bearing more than three levels in which different models can be developed (11, 12). Briefly speaking, RSMs offer two distinctive advantages; simultaneous exploration of factor effects enabling to record interactive effects and requirement for lower trial runs to optimize the process. The latter issue may be very beneficial in saving time and money. Factor levels might be selected upon previous knowledge of the logic numerical or categorical range. Moreover, a quantifiable response is the most important prerequisite to run such statistical designs (design of experiments; DOE).

To our best knowledge, no reports on the application of RSM for evaluating effective parameters on molecular docking accuracy have been reported yet. Our previous work focused on the application of a multifactor RSM analysis to model a docking of fluconazole against various CYP51 conformations with the aim of identifying and ranking significant and interactive effects of computational factors on the docking output of a potent antifungal drug (13). In continuation to our interest in the relevant field (13), we aimed at unveiling the full potential of RSM approaches in optimizing molecular docking simulations with a particular focus of the current study on improving AutoDock4.2 driven binding affinities of citalopram-serotonin transporter (SERT) and donepezil-acetyl cholinesterase (AChE) complexes as prototype systems. 

To address the rationale behind selecting citalopram and donepezil as candidate CNS drugs in this study, a few words are said here regarding the pathophysiology of relevant disorders and the clinical importance of drugs.

Alzheimer’s disease (AD) is the most common neurodegenerative disorder and the sixth most common cause of death in featuring gradually progressive cognitive and functional deficits as well as behavioral changes and is associated with accumulation of amyloid and tau depositions in the brain (14). The cholinergic nervous system and acetylcholinesterase activity are closely related to the pathogenesis of AD. The most used therapy of AD is based on enhancing cholinergic function using inhibitors of acetyl AChE like rivastigmine, donepezil, or galantamine (15). Donepezil is an important oral medication used to improve cognition and behavior in people involved with AD. Depression is a familial mood disorder that causes a persistent feeling of sadness and loss of interest. Also called Major Depressive Disorder (MDD), it affects how you feel, think and behave and can lead to a variety of emotional and physical problems. As it has been predicted that MDD would be the second leading cause of death and disability by the year 2020, it became an ideal target for pharmacogenetic approaches. Among all choices of MDD treatment, the selective serotonin reuptake inhibitor (SSRI) antidepressants such as citalopram are mentioned as the first-line treatment of depression. Genetic variation of SERT is involved in the clinical remission of major depressive episodes after citalopram treatment (16). From the pharmacological aspect of view, candidate targets were selected with regard to the most studied molecular pathways within major disorders worldwide, namely serotonin reuptake inhibition by citalopram (17) and AChE inhibition by donepezil (18) (Table1).

**Figure 1:**
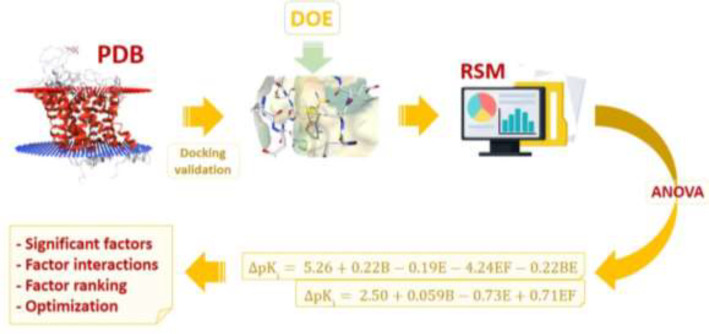
Hierarchical view of the multi-step strategy depicting the application of response surface method (RSM) for molecular docking simulations of CNS drugs citalopram and donepezil into the binding site of validated physiological targets (Serotonin transporter and acetylcholinesterase) retrieved from Brookhaven protein data bank (PDB); Subsequent to target identification, the first step included docking validation for evaluating AutoDock4.2 capability in binding pose prediction. Design of experiments (DOE) for docking trials was performed by Box-Behnken method for six determinant factors; (A) torsion degrees for ligands, (B) grid spacing, (C) quaternion degrees for ligands, (D) No. translation, (E) drug optimization method and (F) target flexibility. Outputs of designed docking trials (Accuracy of target inhibition constant or Δpk_i_) were subjected to analysis of variance (ANOVA) to extract statistical indices and acquire polynomial equation models describing Δpk_i _in association with methodological factors. The final step was dedicated to prioritizing individual and interactive factor effects and numerical optimization to propose enhanced docking simulations

Reference *in-vitro* binding data was retrieved from Binding MOAD, PDB Bind, and Binding DB data banks. For this purpose, candidate drugs were docked inside the binding sites of SERT and AChE according to the Box-Behnken designed matrix. Within the assembled drug matrices, response changes were monitored with simultaneous variations of factor levels, and results were subjected to statistical analysis to produce quadratic models (Figure 1). The final step included numerical optimization with ideal docking accuracies with the aim of achieving enhanced methodological conditions with regard to financial and time restrictions. As it is obvious from the above explanations, the major aim of the current work was to study the effectiveness and accuracy of docking results for a dependent drug-target system and hence not the comparison between drugs. On the basis of this foundation, drugs were not necessarily needed to be chosen from one category since we aimed at numerical optimization of different drug-target interaction systems.

## Experimental

*Drug/target*

Citalopram and donepezil were selected as the candidate drug molecules, and 3D structures of their physiologic targets (SERT and AChE) were retrieved from PDB (www.rcsb.org) with designation codes 5I6X (19), 5I71 (19), 5I73 (19), 4M0E (20), 4EY7 (21) and 5HF9 (22). 

*Molecular docking*

Ligand-flexible molecular docking simulations were performed with Lamarckian Genetic Algorithm (LGA) (23) incorporated into AutoDock 4.2 (7). All the simulation procedures were conducted according to the previous studies (24). Drug-target inhibition constants (K_i_) were estimated through Equations 1 and 2: 

$K_{i}=2.71828\frac{∆G_{b}}{\mathrm{RT}}$

 (1)

${∆}_{G_{b}}=E_{\mathrm{vdW}}+E_{H-\mathrm{bond}}+E_{\mathrm{Desolvation}}+E_{\mathrm{Electrostatic}}+{∆G}_{\mathrm{Torsional}}$

 (2)

In Equation 1, ΔG_b_ represents free binding energy (cal.mol^-1^), R is the gas constant (cal.K^-1^.mol^-1^), and T stands for temperature in kelvin (For docking simulation: 298.15 K), and 2.71828 is indicative of a Napier’s constant. In the case of equation 2, E_vdW_, E_H-bond_, E_Desolvation, _and E_Electrostatic_ represent van der Waals energy, hydrogen bond energy, and desolvation energies for drug-target interaction, and ΔG_Torsional _is the estimated loss of torsional free energy upon binding to the target.

*Experimental design*

All statistical analysis and modeling procedures were performed via the Box-Behnken method incorporated into Design-Expert software-v.7 (State-Ease, Corp., and Minnesota) (25). Methodological factors and their assigned levels to construct models are summarized in Table 2. Three levels were considered for each factor under study. Codes were indicative of low (-1), medium (0) and upper (+1) levels of the factors, respectively. Appropriate factors and their assigned levels were determined in a way that a broad experimental domain within reasonable endpoints could be scanned. 

The subsequent step included the design of experiments (DOE) to offer a Box-Behnken matrix that comprised various docking trials (solutions). Each trial contained different combinations of factor levels. Citalopram and donepezil were docked into the binding sites of SERT and AChE according to DOE trials. A typical matrix for 6 independent factors, each defined in 3 levels, offered 54 docking trials. Results of all docking trials were translated into docking accuracy via Equation 3:

$∆R=\left|R_{\mathrm{Theoretical}}-R_{\mathrm{Experimental}}\right|={\mathrm{pk}}_{\mathrm{i.in silico}}-{\mathrm{pk}}_{\mathrm{i.in vitro}}$

 (3)

R_Theoretical_ is the theoretical response or estimated target inhibitory constant (pk_i,__in-silico_), whereas R_Experimental_ stands for experimental response or *in-vitro* target inhibitory constant (pk_i,__in-vitro_) driven from standard databases (Binding MOAD, PDB Bind and Binding DB). To explain more, the final endpoint was considered an easily detectable parameter pKi, indicative of drug binding affinity. Second-order polynomial functions were used within Equation 4 to correlate the responses with designated factors:

$y=\beta_{0}+\sum_{i=1}^{k}\beta_{i}X_{i}+\sum \beta_{\mathrm{ij}}X_{i}X_{j}+\sum_{i=1}^{k}\beta_{\mathrm{ii}}X_{i}^{2}+\varepsilon$

 (4)

In the above equation, y is the predicted response; β_0_ is an intercept term; βi, βij and βii are linear, quadratic and interaction coefficients, respectively, x_i_ and x_j _are independent variables in coded levels (-1 to 1). The ε value shows a random error. The results were reported by using probability value (*p*-value) with 0.05 as the confidence level. Analysis of variance (ANOVA) was implemented for each endpoint to determine the significant factors of the developed model. The models/factors were recognized as significant in each case if the probability value (*p*-value) was less than 0.05. Model simplifications were carried out via the elimination of non-significant terms (*p* > 0.05) in all of the model equations. Approved models were characterized by F-value, lack of fit F-value, predicted R-squared, adjusted R-squared and Adeq precision to ensure that they could successfully scan the design space.

## Results and Discussion

*Internal validation*

**Figure 2:**
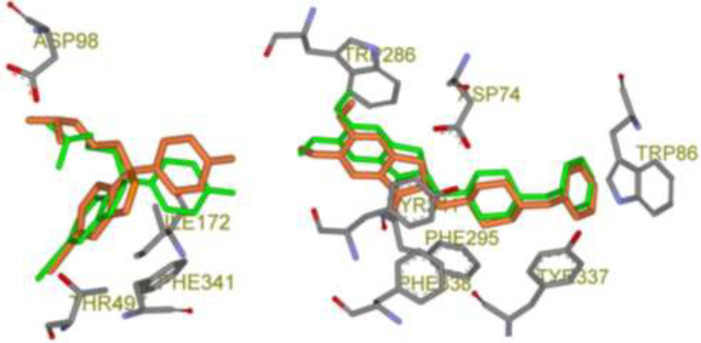
Schematic 3D representation of AutoDock4.2 validation results with key interactive residues of the target, Green stick: Crystallographic pose and Orange stick: Docked pose; Left: Citalopram-AChE complex (PDB code: 5I73, RMSD: 0.82 Å) and Right: Donepezil-SERT complex (PDB code: 4EY7, RMSD: 0.46 Å).

The validity of the AutoDock4.2 method for docking of selected CNS drugs inside their targets was interpreted in terms of RMSD of ligand atoms in re-docked and crystallographic conformations (Table 3). According to the results, all the crystallographic files could pass the filter since AutoDock4.2 could successfully predict the crystallographic (bioactive) conformation (23) within 50 independent GA runs and 2.5 × 106 maximum number evaluations incorporated into LGA. In confirmation of the obtained results, 3D schematic representations of validation results with the best RMSD poses for each drug is depicted in Figure 2.

*Model development and statistical analysis*

**Figure 3:**
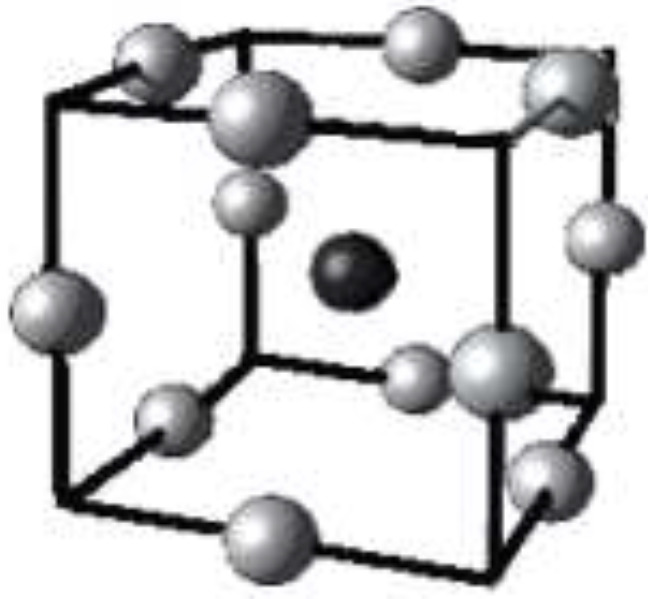
Cubic scheme of Box-Behnken design

Three-level Box-Behnken designs are generated by combining two-level factorial designs and incomplete block designs (26). The technique brings about a few benefits, such as desirable statistical properties and, most importantly, the requirement for only a fraction of trials needed for a 3-level factorial. Run number of Box-Behnken design can be estimated according to Equation 5:

$N=k^{2}+k+\mathrm{cp}$

 (5)

k is the factor number and c*p *is the replicate number of the central point. In a cubic scheme of Box-Behnken design (Figure 3), a model consists of a central point and the middle points of the edges.

*Citalopram*

The ANOVA results for matrix responses (ΔpK_i_ 2.298-4.111) are summarized in Table 4. Statistical analysis proved the quadratic polynomial model to be highly significant (p-value < 0.0001) for data fitting. The acquired model in terms of coded values is illustrated by Equation 6:

$∆pK_{i}=2\cdot 50+0\cdot 059B-0\cdot 059B-0\cdot 73E+0\cdot 71\mathrm{EF}$

 (6)

As referred, for citalopram, the quadratic model was capable of describing the relation between ΔpK_i_ as a dependent variable and factors B (AutoGrid space), E (Drug optimization method), and F (Target flexibility) as independent variables. In the quadratic model, factors and second-order interferences with p-values larger than 0.05 were eliminated by stepwise selection. The lack of fit of the model (1.14) implied that it was not significant with regard to the pure error. Pred R-squared was estimated to be 0.9789, and moreover, it was in reasonable agreement with Adj R-squared (0.9842). A good correlation between factors and responses could be confirmed by the Adj R-squared value, and it meant that most of the variations of response were predictable by model. Adequate precision measures the signal-to-noise ratio and the estimated amount (43.939) was indicative of an adequate signal (A ratio greater than 4 is desirable). On the basis of such model characteristics, it was deduced that obtained model could navigate the design space.

According to ANOVA results (summarized in Table 4), ΔpK_i_ sensitivity to the effective factors could be ranked as E>B>>C>D>A>F, and factors C, D, A & F were detected as insignificant model terms (*p*-value > 0.1). Factors E (drug optimization method) and B (grid spacing) were significant model terms, while factor E (F-value 2204.33) exhibited extremely significant performance. High interactive effects of factors E and F have been observed on the response (*p*-value < 0.0001). Quaternion degrees for drug (factor C) was recognized as an insignificant factor in docking of citalopram into SERT. This indicated that docking accuracy was not dependent on the flipping angle of the citalopram molecule. Lack of significant sensitivity toward variations of factor A (torsion degrees for drug) may be interpreted by the fact that docking simulations are commonly initialized by the co-crystallographic conformations, where fitted binding pose of the drug is applied to run the docking procedure. Such a result may have a practical outcome in docking studies; to achieve a desirable result within reasonable computation times, one might set torsions degrees for the ligand at larger values for more rigid structures.

*Drug optimization method*

Factor E (drug optimization method) was estimated to be the most significant model term in docking of citalopram into ST binding site. The observed highly significant effect might be attributed to the chiral center of citalopram and hence its determinant role in pre-docking conformation of the drug. At first glance, equation 6 indicated that higher docking accuracies could be expected from AM1-based optimization of the citalopram structure method, but due to the highly significant interactive effect of E and F, the best combination toward lowest ΔpK_i_ (2.298) was achieved by PM3 optimization method (coded level of +1). Such a result is in accordance with the inversion barriers of trivalent nitrogen in nitrogen-containing compounds, which are commonly low for AM1 and high for PM3. An apparent consequence is that some nitrogen geometries may be predicted to be flat by AM1 and pyramidal by PM3. Hence it seemed that PM3 could represent a relatively appropriate description of nitrogen geometry and hence more realistic binding interactions with the target.

A common belief is that, unlike ligand-based drug design techniques in which the initial geometry of a bioactive molecule is important, structure-based approaches such as docking are not seriously dependent on the primary optimization of ligand. Indeed beginning a docking practice with a co-crystallographic binding pose of a ligand is a common approach. A rationale is that during molecular docking simulations, molecular conformations are varied via changes in torsion, translation, and quaternion. But the different scenario that was observed with the present study was the highly significant effect of the optimization method (Factor E) on docking output. On the basis of obtained results, it may be assumed that chiral molecules, particularly those bearing nitrogen atoms within a nonpolar scaffold, can undergo an appropriate semi-empirical method such as PM3 to afford better results.

*Target flexibility*

Target flexibility was incorporated into our modeling study via considering different holo-structures of SERT. The results of the statistical analysis were in accordance with what we expected. Higher docking accuracies could be attained via docking of citalopram into the binding site of the SERT structure with the highest resolution (PDB code 5I6X). It was found that decreasing the resolution of SERT from 3.14 Å (5I6X) to 3.24 Å (5I73) reduced docking accuracies (Figure 9). However,, future studies may be directed toward selecting more induced fit models of the protein and statistical analysis through central composite design (CCD techniques). 

*Grid spacing*

A grid map comprises a 3D frame of regularly spaced points for incorporating the target. On the basis of ligand atom types, a probe atom corresponding to the atom type is placed at each grid point, and the energy of interaction of each probe atom (grid point) with surrounding macromolecular atoms is estimated and assigned to the corresponding grid point (24) (Figure 5). Grid spacing (Factor B) is designated as the distance between adjacent grid points. Grid spacing is the distance between adjacent AutoGrid points. ANOVA showed that if the grid spacing is set to lower values, higher AutoDock accuracies for the citalopram-SERT complex will be achieved in confirmation of previous results (13). Lower grid spacings increase the precision of probe scanning within the designated grid box, and this would probably be translated into better SERT inhibition constants. 

*Interactive factor effects*

Factor interaction is likely to occur whenever different responses are generated on the basis of different settings of two factors. This dependence of factor levels to each other may be best interpreted by interaction plots (Figure 6). In this case, interactive factors will be depicted by two non-parallel lines, implying that the effect of one factor depends on the level of the other. ANOVA results proved highly significant interactive effects between factors E (drug optimization method) and F (target flexibility) with *p*-value < 0.0001. 

It was indicated that the significant effect of factor E on estimated SERT inhibition constants was more pronounced at lower levels of F. As could be seen from the graph in Figure 6, the red line is indicative of the effect of the drug optimization method (F) within a SERT 3D structure with PDB code 5I6X and the black line represents the effect of drug optimization method (F) within a SERT 3D structure with PDB code 5I73. Higher docking accuracies might be expected when other factors (A, B, C & D) were held at their lower levels (such as factor F) (Figure 6).

Interaction plots displayed a cross point, and the location of this point showed a distinctive situation within model space in which relatively similar SERT inhibition constants could be expected by docking into all PDB driven 3D SER structures (levels -1 & +1) if the co-crystallographic conformation of citalopram is set as the starting point (Mid-level of factor E).

The 3D surface is known as the “response surface” provided a perspective visualization of factor effects on the response at different levels of other factors. 3D response plots were developed to indicate the simultaneous effect of interactive term EF on docking accuracy (Figure 7). In confirmation of our previous results, a surface was steep and indicated that the interaction between two factors was highly significant. More accurate SERT inhibition constants might be predicted by running the PM3 semi-empirical method (higher levels of factor E) at declined levels of other factors.

*Numerical optimization*

DOE provides a series of solutions (optimum combinations of factor levels) to achieve the most desirable responses. For this purpose, optimization criteria for levels of factors A, B, C, and D were set in range (spanning from -1 to +1) while factors E (drug optimization method) and F (target flexibility) were set at precise levels -1, 0 and +1 since they were categorical but not numerical factors. With the aim of achieving optimized solutions, the goal for response (ΔpK_i_) was primarily set at a minimum.

It should be noted that in each case, solutions with desirability equal to 1 were picked up as optimum. Desirability is an objective function ranging from 0 (worst condition) to 1 (ideal case). This function transforms each response value to a desirability index. The program looks for the largest desirability index and presents a series of solutions that best maximize the desirability index. Obtained results showed that the most accurate predictions of SERT inhibition constant (minimum ΔpK_i_) could be envisaged through various simulations conditions, and careful selection of factors led to highly enhanced accurate responses (Table 5). However, it should be emphasized that choosing the best solution depends on financial and time restrictions. A characteristic feature in all of the proposed docking solutions is the lower level of factor F and a higher level of factor E, which confirmed the previous results of this study.

*Donepezil*

In the case of donepezil, ANOVA results for the responses (ΔpK_i_ 0.240-5.465) are summarized in Table 6. Statistical analysis proved the quadratic polynomial model to be highly significant with a low probability value (*p*-value < 0.0001) for data fitting (Equation 7 in terms of coded values). 

$∆pK_{i}=5\cdot 26+0\cdot 22B-0\cdot 19E-4.24\mathrm{EF}-0\cdot 22\mathrm{BE}$

(7) 

Lack of fit F-value (1.74) implied that lack of fit was not significant with regard to the pure error. There is a 13.36% chance that a “Lack of Fit F-value” this large could occur due to noise. Pred R-squared (0.9820) was in reasonable agreement with Adj R-squared (0.9888). A good correlation between factors and responses could be confirmed by the Adj R-squared value. Adequate precision (43.939) was indicative of an adequate signal. On the basis of obtained data, it was deduced that model could navigate the design space.

According to ANOVA results (summarized in Table 6), factor effects could be ranked as B>E>>D>C>A>F while D, C, A & F were insignificant model terms (*p*-value > 0.1). It was found that factors B (grid spacing) and E (drug optimization method) were significant model terms. Among the pairwise interactions, EF was the significant model term (*p*-value<0.0001) followed by BE (*p*-value 0.0102).

*Grid spacing*

Factor B (grid spacing) was the most significant model term. The polynomial quadratic model (Equation 7) predicted better AChE inhibition constants for donepezil at shorter grid spacings (0.3 Å). The effect was similar to citalopram but more noticeable in the case of donepezil. One possible explanation is the presence of bulky molecular structure of donepezil that necessitates shorter grid spacings in docking simulations.

*Drug optimization method*

Algebraic signs of quadratic model terms (Equation 7) indicated that the application could expect higher docking accuracies of the PM3 method for primary optimization of donepezil structure. Comparative statistical inspection of the results showed that with regard to *p*-values, although being significant, the effect of factor E is more significant for citalopram. This may be attributed to the following explanations:

Unlike citalopram, donepezil includes one nitrogen atom within a hydrophobic structural pattern and inversion barriers of trivalent nitrogen for AM1 and PM3 semi-empirical methods, less dependent on the PM3 optimization method might be explainable.

More flexible structure of citalopram (more active torsions) with regard to donepezil.

*Interactive factor effects*

ANOVA results demonstrated a significant pairwise interactive effect between factors E and F (*p*-value < 0.0001). The significant effect of factor E was most pronounced when donepezil was docked into the AChE model that possessed the highest resolution (PDB 4M0E) (Figure 8). The observed interaction pattern was different from that of citalopram. More accurate enzyme inhibitory activities for donepezil could be expected within two scenarios; AM1-based optimization of drug molecules and docking simulations on 4EY7 or PM3-based optimization of a drug molecule with docking simulations on 4M0E. 

All the interaction plots of EF interactive effects displayed a cross point on mid-levels of factor E (Initial Co-crystallographic conformation of donepezil). To explain more, when co-crystallographic conformation of donepezil was used as the starting point for docking simulations, the estimated AChE inhibition constant was not seriously dependent on the selected PDB model of the target. Such interferences might not be detected via applying one factor at each time method.

One-factor plots confirmed the direction of interactive effects and indicated the highly significant effect of factor E was detected when other factors were held at their upper levels.

3D plots representing simultaneous effects of factors B and E at different levels of other factors are depicted in Figure 10. In upper levels of other factors, more desirable docking accuracies were expected at higher levels of E (PM3 or PM3-like optimization methods). The surface in mid-levels of factors is relatively smooth that was indicative of a less significant interactive effect between B and E.

3D response plots were also developed to interpret the interactive EF effect on docking accuracy. As could be seen from the plots (Figure 11), docking accuracy tended to increase at higher levels of factor E as the levels of other factors declined to lower levels. 3D plots obviously showed that when factor levels were held at their mid-levels, no desirable docking accuracy would be expected. 3D surface plots in lower levels of factors A, C, D and F showed that desirable docking accuracies could be attained at lower levels of factor E and any level of factor B. 

3D response plots were also applied to indicate the simultaneous effect of interactive term EF on docking accuracy (Figure 11). It was found that responses to factor levels fitted a hyperbolic pattern with relative symmetric distribution and steep surfaces. This could be related to the highly significant effect of EF on the response (*p*-value < 0.0001), which is not seriously dependent on the levels of other factors. As could be seen from the plots, more reliable results may be assumed at lower levels of both E and F or higher levels of both E and F. Such interaction pattern can be demonstrated that when higher resolution PDB conformation of AChE is used for docking of donepezil, it would be better to optimize the drug structure with PM3 method while the reverse is true when the lower resolution of AChE conformation is applied. This was also previously confirmed by the interaction plots.

*Numerical optimization*

All the optimization criteria for factors A, B, C, D, E, and F were set as before, and the goal for docking accuracy (ΔpK_i_) was fixed at a minimum. On the basis of offered optimized solutions, maximum docking accuracy (minimum ΔpK_i_) might be achievable via various conditions (Table 7). However, choosing the best solution depends on the financial and time limitations.

In confirmation of ANOVA results, it was revealed that the most accurate predictions of AChE inhibition constant (minimum ΔpK_i_) could be envisaged when both of the factors F and E were set at their upper or lower levels.

**Figure 4:**
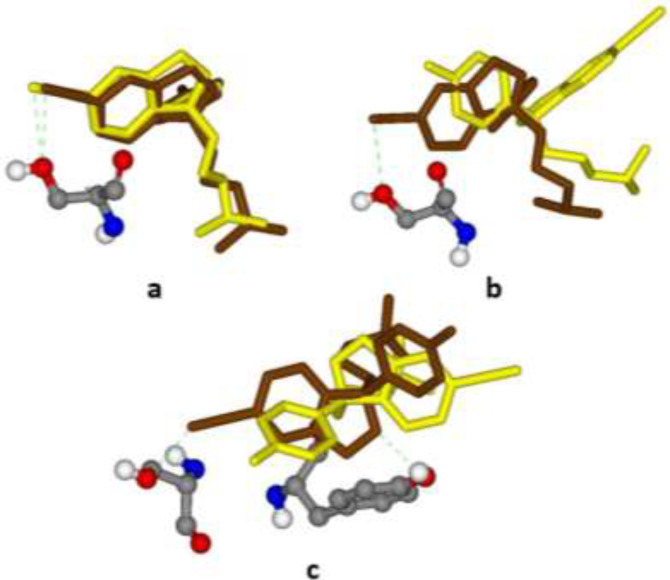
Best (Brown stick) and worst (Yellow stick) binding poses of citalopram within different induced fit models of the serotonin transporter (SERT) along with interacted H-bonds; (a) 5I6X (3.14 Å), Ser439, ΔpK_i_ 2.298-2.536; (b) 5I71 (3.15 Å), Ser439, ΔpK_i_ 2.330-2.697; (c) 5I73 (3.24 Å), Ser439 & Tyr95, ΔpK_i_ 3.850-4.111

**Figure 5:**
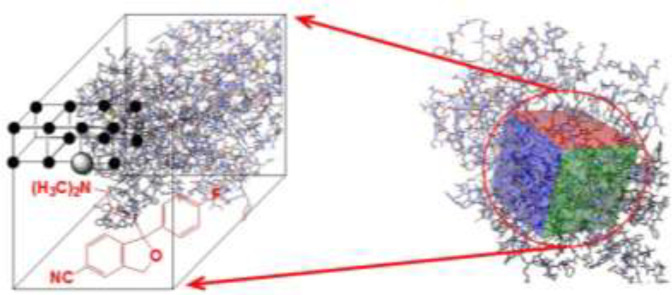
Schematic representation of AutoGrid box and grid points with the larger gray sphere indicating a typical probe atom for the corresponding grid point

**Figure 6:**
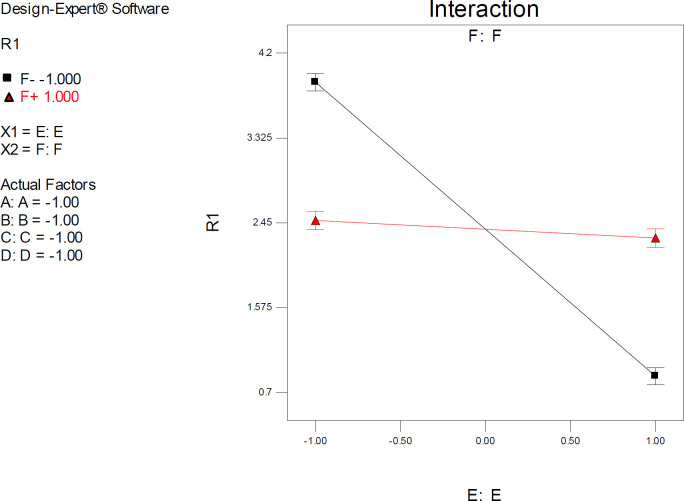
Interaction plot for AutoDock estimated inhibition constants of citalopram-serotonin transporter (SERT) complex representing higher pairwise interaction between factors E (Drug optimization method) and F (Target flexibility) at lower levels of other factors; Red line is indicative of the effect of drug optimization method (F) within a SERT 3D structure with PDB code 5I6X and the black line represents the effect of drug optimization method (F) within a SERT 3D structure with PDB code 5I73**;** R_1_: ΔpK_i_, (A) Torsion degrees for drug, (B) Grid spacing (Å), (C) Quaternion degrees for drug, (D) Translation (Å), (E) Drug optimization method, (F) Target flexibility

**Figure 7:**
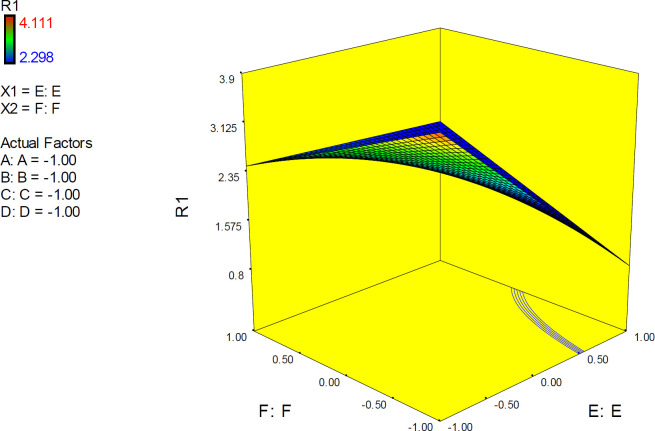
3D surface plot representing the effect of interactive term EF of polynomial quadratic model for AutoDock4.2 driven inhibition constants of citalopram-serotonin transporter (SERT) complex; docking accuracy increased at higher levels of factor E as the levels of other factors declined to lower levels. R_1_: ΔpK_i_ (Docking accuracy), (A) Torsion degrees for drug, (B) Grid spacing (Å), (C) Quaternion degrees for drug, (D) Translation (Å), (E) Drug optimization method, (F) Target flexibility

**Figure 8:**
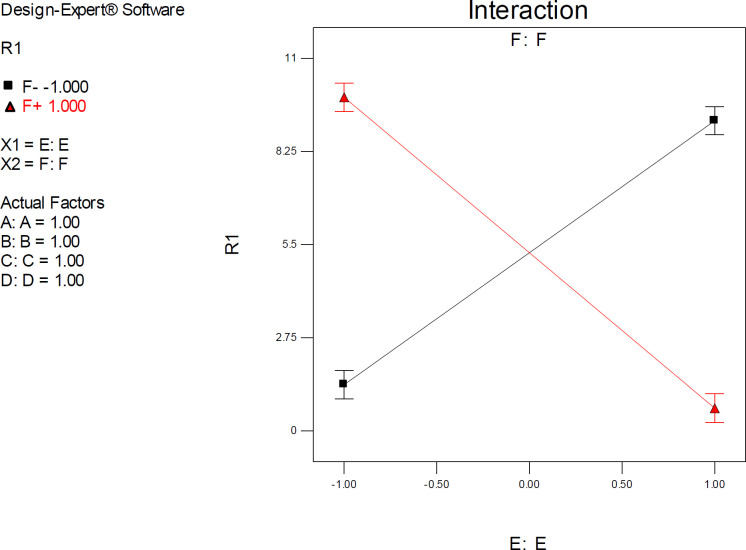
Interaction plot for AutoDock estimated inhibition constants of donepezil-AChE complex representing higher pairwise interaction between factors E (Drug optimization method) and F (Target flexibility) at upper levels of other factors; Red line is indicative of the effect of drug optimization method (F) within a SERT 3D structure with PDB code 4M0E and the black line represents the effect of drug optimization method (F) within an AChE 3D structure with PDB code 4EY7; R_1_: ΔpK_i_, (A) Torsion degrees for drug, (B) Grid spacing (Å), (C) Quaternion degrees for drug, (D) Translation (Å), (E) Drug optimization method, F: Target flexibility

**Figure 9:**
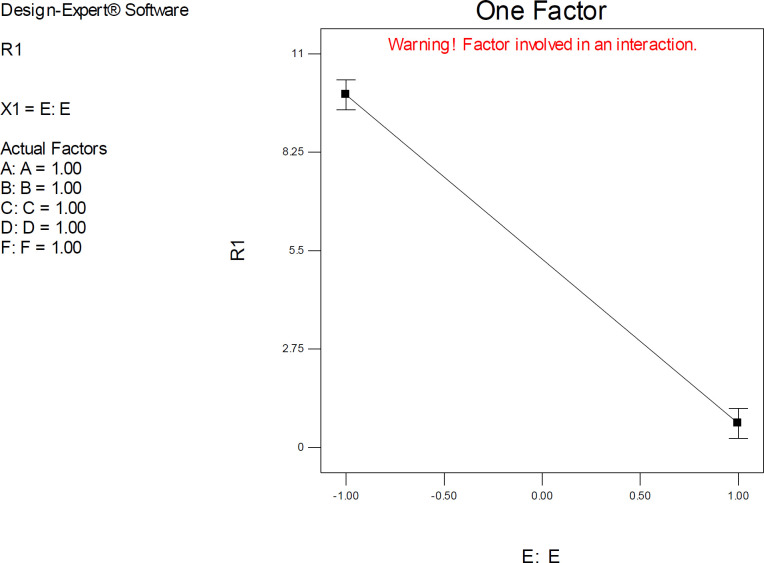
The one-factor plot of AutoDock estimated inhibition constants of donepezil-AChE complex representing the higher effect of factors E (Drug optimization method) at upper levels of other factors; R_1_: ΔpK_i_ (Docking accuracy), (A) Torsion degrees for drug, (B) Grid spacing (Å), (C) Quaternion degrees for drug, (D) Translation (Å), (E) Drug optimization method, (F) Target flexibility

**Figure 10:**
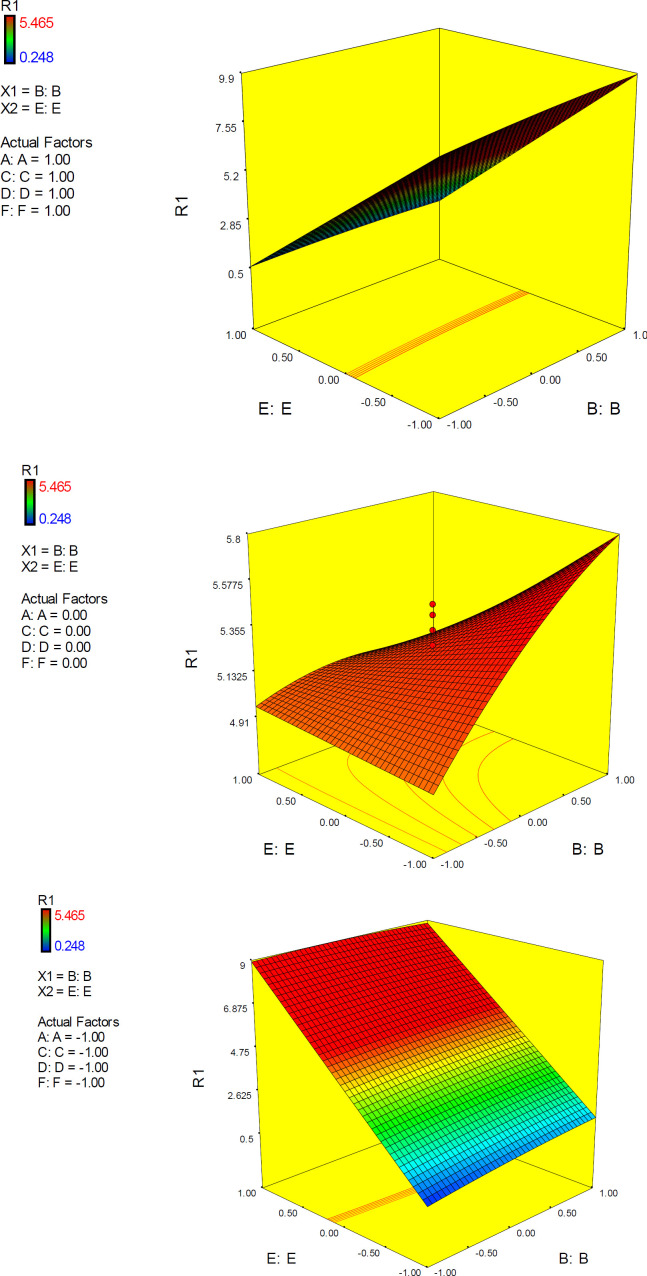
3D surface plot representing the effect of interactive term BE of polynomial quadratic model for AutoDock4.2 driven inhibition constants of donepezil-AChE complex; R_1_: ΔpK_i_ (Docking accuracy), (A) Torsion degrees for drug, (B) Grid spacing (Å), (C) Quaternion degrees for drug, (D) Translation (Å), (E) Drug optimization method, (F) Target flexibility

**Figure 11:**
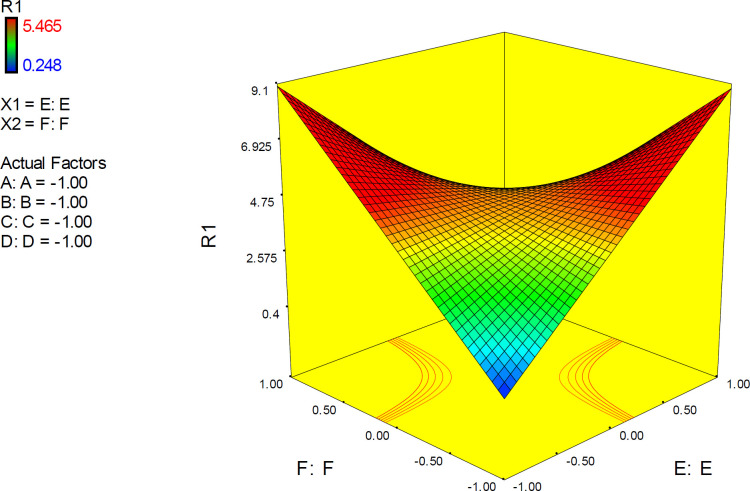
3D surface plot representing the effect of interactive term EF of polynomial quadratic model for AutoDock4.2 driven inhibition constants of donepezil-AChE complex; docking accuracy increased at higher levels of factor E as the levels of other factors declined to lower levels. (A) Torsion degrees for drug, (B) Grid spacing (Å), (C) Quaternion degrees for drug, (D) Translation (Å), (E) Drug optimization method, (F) Target flexibility

**Table 1:** Characteristics of candidate CNS drugs and their mechanisms of pharmacological action

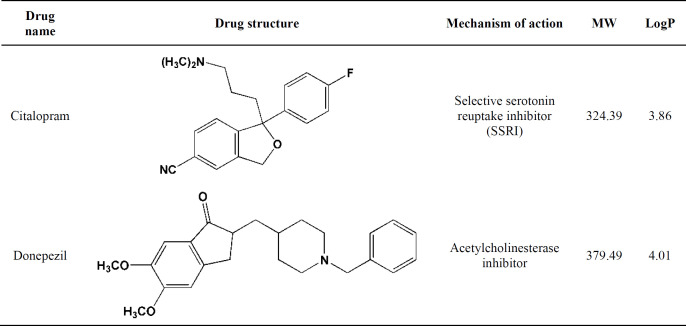

**Table 2:** Actual/coded values of selected factors for AutoDock4.2 based RSM study of citalopram and donepezil

**Factors understudy**	**Factor levels**		
	**Low: Actual (Coded)**	**Medium: Actual (Coded)**	**High: Actual (Coded)**
A: Torsion degrees for drug	5 (-1)	20 (0)	50 (+1)
B: Grid spacing (Å)	0.3 (-1)	0.375 (0)	0.5 (+1)
C: Quaternion degrees for drug	5 (-1)	20 (0)	50 (+1)
D: Translation (Å)	0.2 (-1)	0.3 (0)	0.5 (+1)
E: Drug optimization method	AM1 (-1)	Cognate ligand (0)	PM3 (+1)
F: Target flexibility	Lowest resolution (Å) PDB code (-1)	Medium resolution (Å) PDB code (0)	Highest resolution (Å) PDB code (+1)

**Table 3:** AutoDock 4.2 validation results for different *holo* PDB structures of intended CNS targets

**Drug/No.**	**PDB ID**	**Resolution of** **PDB structure** **(Å)**	**Number of GA runs**	**No. of conformationsin top-ranked cluster**	**Maximum** **No. of energy eval.**	**RMSD from Reference (Å)**
**Citalopram**						
1	5I6X	3.14	50	49	2.5 × 10^6^	1.58
2	5I71	3.15	50	49	2.5 × 10^6^	0.90
3	5I73	3.24	50	50	2.5 × 10^6^	0.82
**Donepezil**						
1	4M0E	2.00	50	50	2.5 × 10^6^	0.55
2	5HF9	2.20	50	50	2.5 × 10^6^	1.92
3	4EY7	2.35	50	47	2.5 × 10^6^	0.46

**Table 4:** ANOVA report for significant terms of a polynomial quadratic model in docking study of citalopram-serotonin transporter complex

**Source**	**Sum of squares**	**df**	**Mean square**	**F-value**	***P*** **-value**
Model	19.41	11	1.76	301.22	<0.0001
B	0.082	1	0.082	14.02	0.0005
E	12.91	1	12.91	2204.33	<0.0001
EF	5.59	1	5.59	954.33	<0.0001
Residual	0.25	42	5.858E-003		
Lack of fit	0.15	25	6.160E-003	1.14	0.3987
Pure error	0.092	17	5.415E-003		
Cor Total	19.66	53			

**Table 5:** RSM-based optimum solutions for AutoDock4.2 simulations (in terms of coded factor levels) leading to the most accurate inhibition constants of serotonin transporter by citalopram (1 > ΔpK_i_); A: Torsion degrees for drug; B: Grid spacing (Å); C: Quaternion degrees for drug; D: Translation (Å); E: Drug optimization method; F: Target flexibility

**No. optimized solution**	**Factor levels**						
	**A**	**B**	**C**	**D**	**E**	**F**	**ΔpK**_i_
1	-0.33	-0.96	-0.85	0.14	1.00	-1.00	0.925
2	0.42	-0.79	-0.67	-0.50	1.00	-1.00	0.933
3	-0.25	-0.73	-0.57	-0.85	1.00	-1.00	0.934
4	0.42	-0.40	-0.90	-0.85	1.00	-1.00	0.938
5	-0.97	-0.41	-0.78	0.04	1.00	-1.00	0.960
6	-0.85	-0.93	0.67	-0.27	1.00	-1.00	0.968
7	-0.69	-0.94	-0.36	0.84	1.00	-1.00	0.987
8	-0.32	-0.40	-0.04	-0.73	1.00	-1.00	0.996
9	-0.70	-0.50	0.28	-0.32	1.00	-1.00	0.999

**Table 6:** ANOVA results for significant terms of a polynomial quadratic model in docking study of donepezil-AChE complex

**Source**	**Sum of squares**	**df**	**Mean square**	**F-value**	***P*** **-value**
Model	248.19	20	12.41	234.38	<0.0001
B	1.19	1	1.19	22.42	<0.0001
E	0.91	1	0.91	17.18	0.0002
BE	0.39	1	0.39	7.42	0.0102
EF	189.12	1	189.12	3571.88	<0.0001
Residual	1.75	33	0.053		
Lack of fit	1.08	16	0.068	1.74	0.1336
Pure error	0.66	17	0.039		
Cor Total	249.94	53			

**Table 7:** RSM-based optimum solutions for AutoDock4.2 simulations (in terms of coded factor levels) leading to the most accurate inhibition constants of AChE by citalopram (0.2 > ΔpK_i_); A: Torsion degrees for drug; B: Grid spacing (Å); C: Quaternion degrees for drug; D: Translation (Å); E: Drug optimization method; F: Target flexibility

**No. Optimized solution**	**Factor levels**						
	**A**	**B**	**C**	**D**	**E**	**F**	**ΔpK**_i_
1	-0.45	-0.99	0.93	-0.97	1.00	1.00	0.152
2	0.99	-1.00	-0.90	0.99	-1.00	-1.00	0.160
3	-0.06	-0.86	1.00	-0.91	1.00	1.00	0.161
4	-0.08	-0.49	1.00	-1.00	1.00	1.00	0.187
5	-0.11	-0.89	0.98	-0.96	-1.00	-1.00	0.185

## Conclusion

Availability of facile, time-efficient, and accurate computer-aided or *in-silico* drug design techniques is an urgent requirement for identifying and developing potent and selective medicinal agents. Within the structure-based strategies, molecular docking is a frequently used and valuable computational method for matching ligands/drugs into the environment of a validated target. Despite several advantages and fruitful historical outcomes, current docking simulations are mostly restricted to inaccurate estimated binding affinities. In light of the above explanations, improving docking accuracy to fill the gap between theoretical and experimental data through statistical optimization of effective variables may be plausible. Efficient statistical techniques such as RSM can be appropriately utilized for the identification of effective factors and their optimization toward more robust docking simulations. RSMs offers a substantial advantage over commonly applied one-factor-at-each-time techniques in a way that, besides individual factor effects, interactive effects may also be considered within noticeably fewer trials. Within the present contribution, the full potential of RSM in optimizing molecular docking simulations was unveiled through Box-Behnken derived ANOVA analysis of AutoDock4.2 based binding affinity prediction. For this purpose, polynomial, quadratic models were constructed for the binding of highly prescribed antidepressant (citalopram) (R^2^ 0.9789) and anti-Alzheimer’s (donepezil) (R^2^ 0.9820) drugs to physiological targets SERT and AChE. Significant individual and interactive factor effects on the accuracy of estimated target inhibition constants were statistically elucidated. It was revealed that estimated binding affinities citalopram and donepezil were mostly affected by the pre-docking optimization method and AutoGrid spacing, respectively. One of the advantageous features of RSMs is the identification of interactive effects that simultaneously change the response. For citalopram, the optimization method exhibited significant pairwise interaction with conformational flexibility of SERT, while in the case of donepezil, the binding of the drug to AChE was significantly affected by the interactive effect of grid spacing with the optimization method. Probably the most productive section of study results was the numerical optimization that offered a few optimized docking simulations leading to significantly higher accuracies in AutoDock4.2 driven SERT and AChE inhibition constants. The outputs of this study may indicate the full potential of RSMs for the development of optimized AutoDock protocols toward the rational design of privileged medicinal scaffolds.
